# Supplementary material for: Clonal kinetics and single-cell transcriptional profiling of CAR-T cells in patients undergoing CD19 CAR-T immunotherapy
Source: Nat Commun. 2020 Jan 10;11:219. doi: 10.1038/s41467-019-13880-1 (PMC6954177; doi:10.1038/s41467-019-13880-1)
Supplement: Supplementary file 1 — Supplementary Information [file 41467_2019_13880_MOESM1_ESM.pdf]

Clonal kinetics and single-cell transcriptional profiling of CAR-T cells in patients undergoing CD19 CAR-T immunotherapy

Sheih *et al.*

Supplementary Material (Figures and Tables)

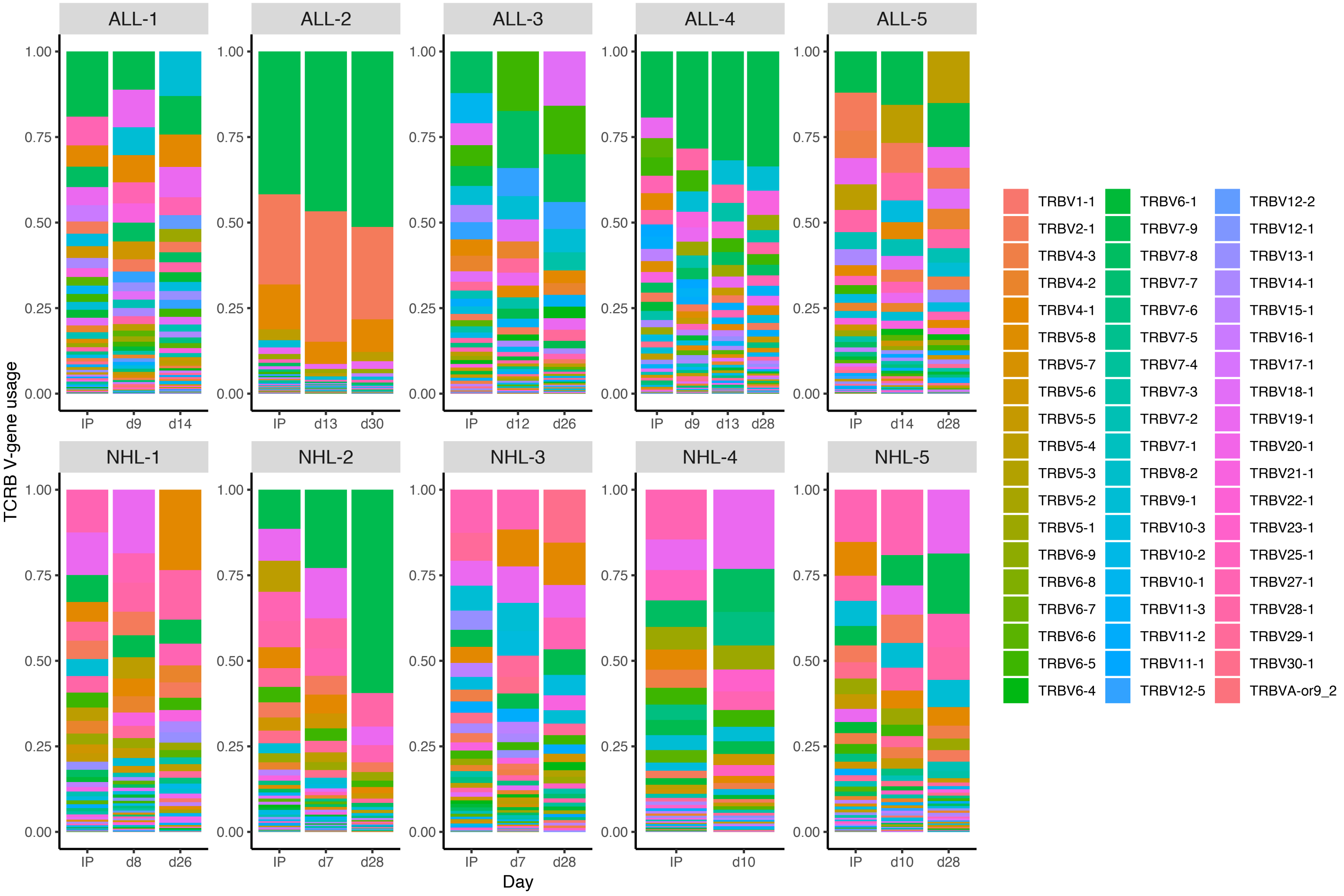

Supplementary Figure 1. Multiple TCRB gene families are represented in CD8⁺ CAR-T cells in the infusion product and isolated at the early and late time points after infusion. Bars depict the proportion of CD8⁺ CAR-T cells belonging to the indicated TCRB family. Source data are provided as a Source Data file.

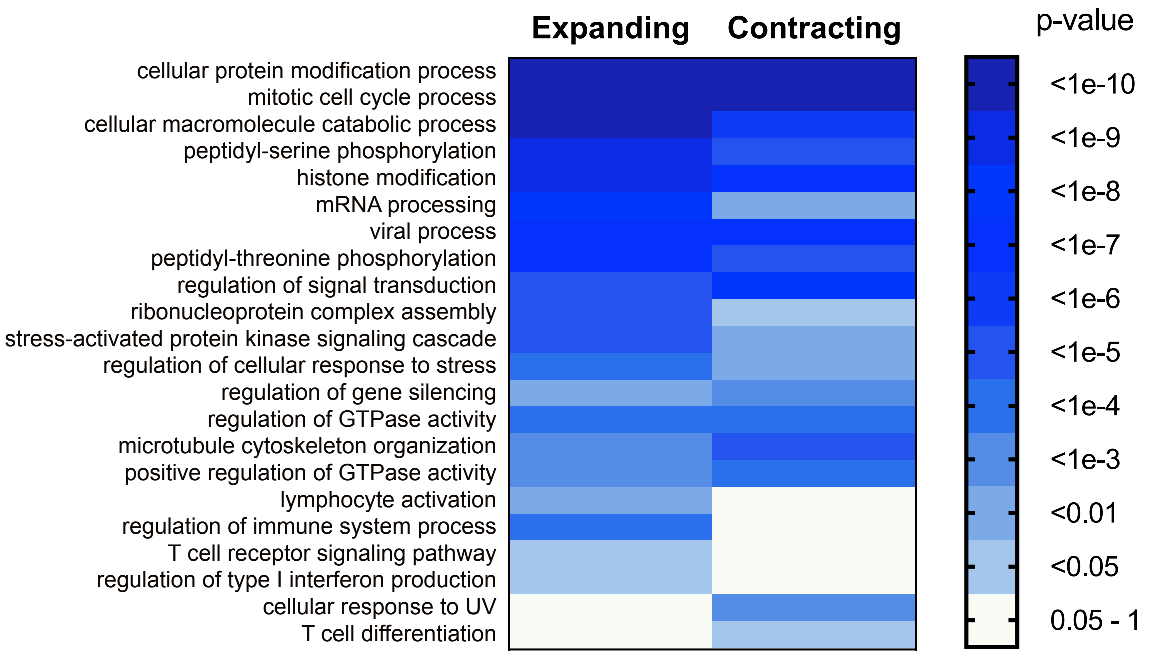

Supplementary Figure 2. Heatmap of biological processes identified using Gene Ontology (GO) enrichment analysis. Integration sites that either increased (Expanding) or decreased (Contracting) in relative abundance by at least 5-fold between the infusion product and in blood after adoptive transfer were identified and combined across all patients. The most significantly enriched GO categories in either expanding or contracting integration sites are plotted as a row of individual boxes and color coded based on FDR adjusted p-value. Source data are provided as a Source Data file.

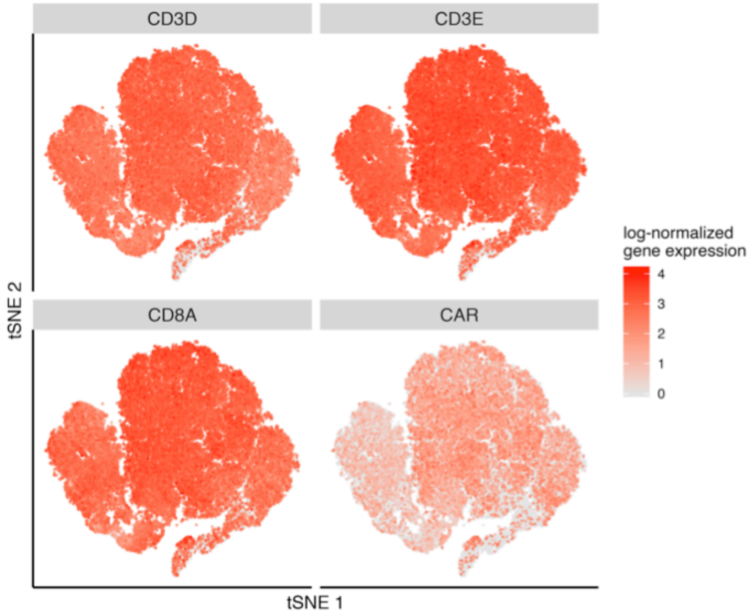

Supplementary Figure 3. t-SNE plot of concatenated CD8$^+$ CAR-T cells from IP, early, late, and very late time point shows log-normalized gene expression of *CD3D*, *CD3E*, *CD8A*, and scFv of the CD19 CAR construct. The scRNA-seq method only sequences and aligns the 5' end of the mRNA transcript. Given that the CAR construct can randomly integrate into the genome, this method might not detect all scFv sequences of the CD19 CAR.

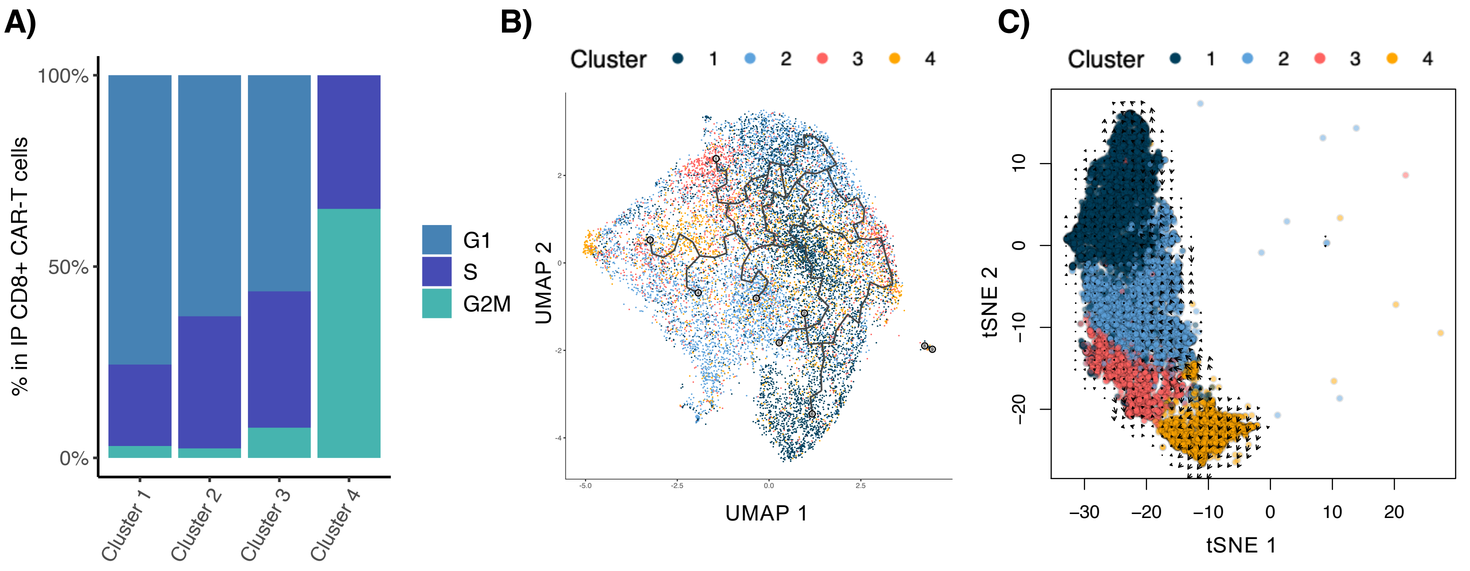

Supplementary Figure 4. (A) The percentage of CD8⁺ CAR-T cells within each infusion product cluster that is predicted to be in the G1, S, or G2M phase. (B) CD8⁺ CAR-T cells in the infusion product of all four patients were analyzed using Monocle 3 and colored based on cluster assignment. Pseudotime analysis was performed after correcting the dataset for cell cycle scores, nUMI, and the percentage of mitochondrial genes. The defined trajectories are shown as black line segments. (C) RNA velocity of CD8⁺ CAR-T cells in the infusion product were computed and visualized on t-SNE plot. Arrows indicate the direction of the future states.

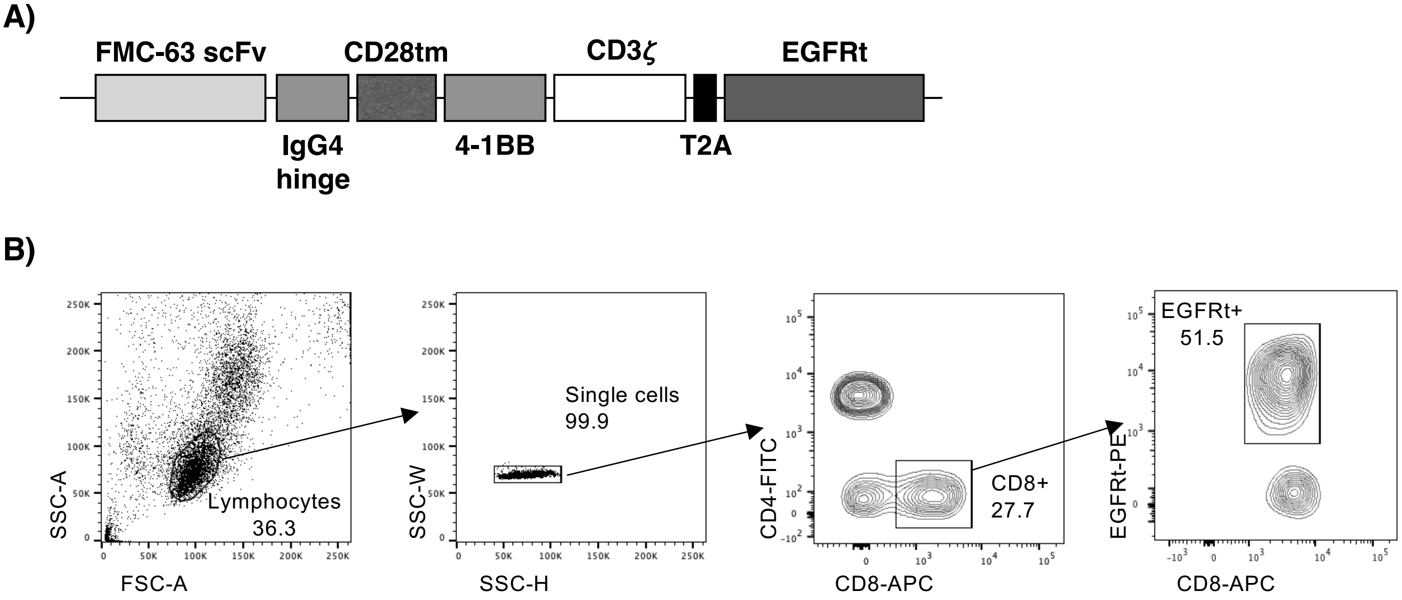

Supplementary Figure 5. (A) CAR construct comprising an FMC63-derived scFv, an IgG4 hinge spacer, a CD28 transmembrane domain, and 4-1BB costimulatory and CD3ζ signaling domains, separated by a ribosomal skip sequence from a truncated human epidermal growth factor receptor (EGFRt). (B) Flow cytometry gating strategy for isolation of $CD8^+$ CAR-T cells from the infusion product and aliquots of PBMCs obtained from blood of patients after infusion. This gating strategy was used to isolate CD8+ CAR-T cells for TCRB sequencing and single-cell RNA sequencing.

Supplementary Table 1: Patient Data and Treatment Characteristics

ID	Assay	Age	Sex	CD8 T cell selection	LD	Cell dose (EGFRt^{+} cells/kg)	Neurotox	CRS	Outcome	DFS/PFS Status	DFS/PFS Day
ALL-1	T	37	F	CD8TCM	Cy	$2x10^5$	0	2	CR	Relapse	66
ALL-2	T, I	62	F	CD8TCM	Cy	$2x10^5$	0	2	CR	Relapse	65
ALL-3	T, I	40	F	CD8TCM	Cy/Flu	$2x10^6$	0	2	CR	CR Ongoing	1098
ALL-4	T, I	22	M	CD8TCM	Cy/Flu	$2x10^6$	3	2	CR	CR Ongoing	765
ALL-5	T	24	F	CD8TCM	Cy	$2x10^5$	3	3	CR	Relapse	126
NHL-1	T	65	M	CD8TCM	Cy/Flu	$2x10^6$	0	0	CR	Relapse	298
NHL-2	T, I	36	M	CD8TCM	Cy/Flu	$2x10^7$	0	2	PR	Progression	58
NHL-3	T, I	51	M	CD8TCM	Cy/Flu	$2x10^7$	0	2	CR	Progression	76
NHL-4	T	57	M	CD8TCM	Cy/E	$2x10^5$	0	2	PR	Progression	41
NHL-5	T	68	M	CD8TCM	Cy/E	$2x10^6$	0	1	PR	Relapse	274
NHL-6	10x, I	64	M	CD8TCM	Cy/Flu	$2x10^6$	3	4	CR	Progression	285
NHL-7	10x, I	51	F	CD8TCM	Cy/Flu	$2x10^6$	1	0	CR	CR Ongoing	393
CLL-1	10x	61	M	CD8	Cy/Flu	$2x10^5$	0	0	CR	CR Ongoing	947
CLL-2	10x	53	F	CD8	Cy/Flu	$2x10^6$	3	2	CR	CR Ongoing	764

Abbreviations: ALL, acute lymphoblastic leukemia; NHL, non-Hodgkin lymphoma; CLL, chronic lymphocytic leukemia; CD8, CD8^{+} selection; CD8TCM, CD8^{+} central memory selection; LD, lymphodepletion; Cy, cyclophosphamide; Flu, fludarabine; E, ectoposide; CRS, cytokine release syndrome; CR, complete response; PR, partial response; DFS, disease free survival; PFS, progression free survival. Assay: T = TCRB sequencing, I = Integration site analysis, 10x = 10x single-cell RNA-sequencing

Supplementary Table 2: Summary of TCRB gene sequencing results show the number of total sequences, productive sequences, unique sequences, and unique productive sequences in each sample.

Patient ID	Time points	# total sequence	# productive sequence	# unique sequence	# unique productive sequence
ALL-1	IP	607,127	473,848	3,878	2,886
ALL-1	d+9	72,416	50,163	684	450
ALL-1	d+14	200,649	139,547	1,025	687
ALL-2	IP	1,053,016	964,806	1,951	1,442
ALL-2	d+13	641,790	619,507	986	693
ALL-2	d+30	632,432	604,522	726	537
ALL-3	IP	4,066,871	2,900,108	6,463	4,687
ALL-3	d+12	1,103,437	741,188	1,780	1,218
ALL-3	d+26	1,527,475	992,531	1,784	1,198
ALL-4	IP	1,609,425	1,275,942	3,367	2,409
ALL-4	d+9	1,355,112	1,122,364	2,112	1,458
ALL-4	d+13	221,716	172,460	814	517
ALL-4	d+28	339,060	269,378	874	549
ALL-5	IP	679,436	537,441	3,325	2,524
ALL-5	d+14	126,228	100,344	867	623
ALL-5	d+28	112,494	84,833	670	425
NHL-1	IP	3,450,345	2,726,823	13,262	10,313
NHL-1	d+8	11,949	7,403	442	262
NHL-1	d+26	19,880	9,164	344	130
NHL-2	IP	2,036,531	1,662,817	11,524	9,171
NHL-2	d+7	208,473	177,457	1,507	1,067
NHL-2	d+28	210,127	180,691	812	516
NHL-3	IP	2,494,719	2,017,132	6,391	4,923
NHL-3	d+7	100,134	76,748	887	575
NHL-3	d+28	6,839	3,234	250	93
NHL-4	IP	855,310	664,541	2,889	2,271
NHL-4	d+10	673,456	531,125	1,113	829
NHL-5	IP	1,754,834	1,292,267	5,923	4,464
NHL-5	d+10	348,178	237,196	778	546
NHL-5	d+28	942,357	586,423	1,641	1,119

Supplementary Table 3: Gene sets used for gene set enrichment analysis in transcriptional profiling of single CD8$^+$ CAR-T cells

BTM - T cell activation (II)	KEGG - Citrate Cycle TCA Cycle	KEGG - Oxidative Phosphorylation	Tem/eff>Tn/Tcm (Willinger 2005)	Exhaustion (Wherry 2007)	Exhaustion (Long 2015)
CCL5	ACLY	ATP12A	KLRF1	TNFSF6	KIAA1324
SP140	ACO1	ATP4A	SLCO4C1	PBX3	FN1
TIGIT	ACO2	ATP4B	CX3CR1	GP49B	ITGA9
CD247	CS	ATP5A1	CD160	CD244	SMPDL3A
CCR5	DLAT	ATP5B	GZMA	CCL3	ITPRIPL2
NLRC3	DLD	ATP5C1	CD244	EOMES	PRSS23
PTPRCAP	DLST	ATP5D	HLA-DRA	CASP3	CLECL1
STAT4	FH	ATP5E	PTGDR	PLSCR1	GRB10
IL12RB1	IDH1	ATP5F1	CCL4	KDT1	KIF21A
TRAT1	IDH2	ATP5G1	PRF1	CTLA4	CTLA4
SLA2	IDH3A	ATP5G1P5	CHST12	PDCD1	ATP10D
CXCR3	IDH3B	ATP5G2	KLRD1	IER5	NUDT16
ZAP70	IDH3G	ATP5G3	PTGDS	RGS16	PLD1
NKG7	LOC283398	ATP5H	APOBEC3G	A430109M19RIK	LGMN
SIRPG	LOC642502	ATP5I	KLRA1	TNFRSF9	SASH1
ICOS	MDH1	ATP5J	CD300A	PENK1	ZNF704
IFNG	MDH2	ATP5J2	NKG7	EOMES	PLEKHH2
IL18R1	OGDH	ATP5L	FGR	COCH	CTHRC1
SLAMF7	OGDHL	ATP5O	PLCG2	PTPN13	ID1
PTPN7	PC	ATP6	CTSC	TCRG-V4	FAXC
ITK	PCK1	ATP6AP1	CST7	NR4A2	EGR1
CRTAM	PCK2	ATP6V0A1	PTGER2	CD160	TNFSF11
GZMA	PDHA1	ATP6V0A2	HLA-DPA1	PTGER4	PTGIS
CD3E	PDHA2	ATP6V0A4	HLA-DRB1	CCL4	KLRC1/KLRC2
GPR171	PDHB	ATP6V0B	CD58	WBP5	CD38
TARP	SDHA	ATP6V0C	DMN	GPR56	RBM47
CD3D	SDHB	ATP6V0D1	GZMK	1110067D22RIK	SPINK2
LCK	SDHC	ATP6V0D2	GOLPH3L	ENTPD1	AKAP5
SLAMF1	SDHD	ATP6V0E1	TNFRSF9	SH2D2A	ENTPD1
CD3G	SUCLA2	ATP6V0E2	KIF11	SEPT4	
	SUCLG1	ATP6V1A	AOAH	ISG20	
	SUCLG2	ATP6V1B1	POU2AF1	TRIM47	
		ATP6V1B2	RRBP1	SERPINA3G	
		ATP6V1C1	FLJ14213	CASP4	
		ATP6V1C2	VCAM1	9130009C22RIK	
		ATP6V1D	JAKMIP2	C79248	
		ATP6V1E1	FLJ11151	LAG3	
		ATP6V1E2	HLA-DQA1	NR4A2	
		ATP6V1F	SMC2	NFTAC1	
		ATP6V1G1	CTNNA1	CAR2	
		ATP6V1G2	CRTAM	C330007P06RIK	
		ATP6V1G3		GPD2	
		ATP6V1H		2700084L22RIK	
		ATP8		RNF11	
		COX1		CAPZB	
		COX10		TUBB2	
		COX11		BUB1	
		COX15		JAK3	
		COX17		9130410M22RIK	
		COX2		CD9	

BTM - T cell activation (II)	KEGG - Citrate Cycle TCA Cycle	KEGG - Oxidative Phosphorylation	Tem/eff>Tn/Tcm (Willinger 2005)	Exhaustion (Wherry 2007)	Exhaustion (Long 2015)
		COX3		TCRG-V4	
		COX4I1		1810054D07RIK	
		COX4I2		RCN	
		COX5A		2010100O12RIK	
		COX5B		SYBL1	
		COX6A1		ETF1	
		COX6A2		CPA3	
		COX6B1		CD7	
		COX6B2		ART3	
		COX6C		1810035L17RIK	
		COX6CP3		ATF1	
		COX7A1		PRKWNK1	
		COX7A2		MTV43	
		COX7A2L		CIT	
		COX7B		CCRL2	
		COX7B2		ADFP	
		COX7C		D8ERTD531E	
		COX8A		TCEA2	
		COX8C		MYH4	
		CYC1		TNFRSF1A	
		CYTB		SPP1	
		LHPP		S100A13	
		LOC100133737		PON2	
		LOC642502		AI181996	
		LOC644310		G1P2	
		LOC727947		TANK	
		ND1		SHKBP1	
		ND2		2510004L0RIK	
		ND3		D15ERTD781E	
		ND4		ICSBP1	
		ND4L		BC024955	
		ND5		GDF3	
		ND6		ITGAV	
		NDUFA1		1110006I15RIK	
		NDUFA10		CPSF2	
		NDUFA11		KLK6	
		NDUFA2		CPT2	
		NDUFA3		LMAN2	
		NDUFA4		TOR3A	
		NDUFA4L2		CRYGB	
		NDUFA5		GPR65	
		NDUFA6		MKI67	
		NDUFA7		TCRB-V13	
		NDUFA8		NPTXR	
		NDUFA9		SNRPB2	
		NDUFAB1		NDFIP1	
		NDUFB1		PTGER2	
		NDUFB10		ZFP91	
		NDUFB2		SPOCK2	
		NDUFB3		5730469M10RIK	
		NDUFB4		CXCL10	
		NDUFB5		GCIN	
		NDUFB6		TRIM25	
		NDUFB7		WBSCR5	
		NDUFB8		MOX2	

BTM - T cell activation (II)	KEGG - Citrate Cycle TCA Cycle	KEGG - Oxidative Phosphorylation	Tem/eff>Tn/Tcm (Willinger 2005)	Exhaustion (Wherry 2007)	Exhaustion (Long 2015)
		NDUFB9		DOCK7	
		NDUFC1		PAWR	
		NDUFC2		CHL1	
		NDUFS1			
		NDUFS2			
		NDUFS3			
		NDUFS4			
		NDUFS5			
		NDUFS6			
		NDUFS7			
		NDUFS8			
		NDUFV1			
		NDUFV2			
		NDUFV3			
		PPA1			
		PPA2			
		SDHA			
		SDHB			
		SDHC			
		SDHD			
		TCIRG1			
		UQCR10			
		UQCR11			
		UQCRB			
		UQCRC1			
		UQCRC2			
		UQCRFS1			
		UQCRH			
		UQCRHL			
		UQCRQ			

Supplementary Table 4: Number of IRF/DRF clonotypes and CAR-T cells identified in NHL-6 and NHL-7

	IRF Clonotypes	IRF CAR-T cells	DRF Clonotypes	DRF CAR-T cells
NHL-6	29	322	67	1187
NHL-7	19	40	59	1447

IRF: Increased relative frequency in blood compared to the infused product
DRF: Decreased relative frequency in blood compared to the infused product

IRF clonotypes were identified as clones in the IP whose relative frequency at the early time point was significantly higher compared to the relative frequency in the IP (Fisher's exact test, FDR of 5%). DRF clonotypes were identified as clones in the IP whose relative frequency at the early time point was significantly lower compared to the relative frequency in the IP (Fisher's exact test, FDR of 5%).

Supplementary Table 5: Primers used in amplification of integration loci

Oligonucleotide	Sequence
Lentivirus LTR Primer (1st PCR)	5’-TCGTCGGCAGCGTCAGATGTGTATAAGAGACAGAGCTTGCCTTGAGTGCTTCAAGTAG-3’
Lentivirus LTR Primer (2nd PCR)	5’-[Sample-specific Barcode]-AGTAGTGTGTGCCCGTCTGT-3’
Linker Cassette Sense	5`-GACCCGGGAGATCTGAATTCAGTGGCACAGCAGTTAGG-3`
Linker Cassette Antisense	5`-CCTAACTGCTGTGCCACTGAATTCAGATC-3`
Linker Cassette Primer	5’-GTCTCGTGGGCTCGGAGATGTGTATAAGAGACAGGATCTGAATTCAGTGGCACAG-3’
