# Supplementary material for: Clonal kinetics and single-cell transcriptional profiling of CAR-T cells in patients undergoing CD19 CAR-T immunotherapy
Source: Nat Commun. 2020 Jan 10;11:219. doi: 10.1038/s41467-019-13880-1 (PMC6954177; doi:10.1038/s41467-019-13880-1)
Supplement: Supplementary file 3 — Reporting Summary [file 41467_2019_13880_MOESM3_ESM.pdf]

Corresponding author(s): Cameron Turtle
Last updated by author(s): Nov 22, 2019

Reporting Summary

Nature Research wishes to improve the reproducibility of the work that we publish. This form provides structure for consistency and transparency in reporting. For further information on Nature Research policies, see Authors & Referees and the Editorial Policy Checklist.

Statistics

For all statistical analyses, confirm that the following items are present in the figure legend, table legend, main text, or Methods section.

n/a	Confirmed	
☐	☒	The exact sample size (*n*) for each experimental group/condition, given as a discrete number and unit of measurement
☐	☒	A statement on whether measurements were taken from distinct samples or whether the same sample was measured repeatedly
☐	☒	The statistical test(s) used AND whether they are one- or two-sided<br>*Only common tests should be described solely by name; describe more complex techniques in the Methods section.*
☒	☐	A description of all covariates tested
☐	☒	A description of any assumptions or corrections, such as tests of normality and adjustment for multiple comparisons
☐	☒	A full description of the statistical parameters including central tendency (e.g. means) or other basic estimates (e.g. regression coefficient) AND variation (e.g. standard deviation) or associated estimates of uncertainty (e.g. confidence intervals)
☐	☒	For null hypothesis testing, the test statistic (e.g. F, t, r) with confidence intervals, effect sizes, degrees of freedom and P value noted<br>*Give P values as exact values whenever suitable.*
☒	☐	For Bayesian analysis, information on the choice of priors and Markov chain Monte Carlo settings
☒	☐	For hierarchical and complex designs, identification of the appropriate level for tests and full reporting of outcomes
☒	☐	Estimates of effect sizes (e.g. Cohen's d, Pearson's r), indicating how they were calculated

Our web collection on statistics for biologists contains articles on many of the points above.

Software and code

Policy information about availability of computer code

Data collection	The Cell Ranger Single-Cell Software Suite (version 2.1.0) was used to initially process the single-cell RNA-sequencing data.<br>For integration site sequencing:<br>Agilent 2200 TapeStation<br>Life Technologies Qubit 2.0 fluoromter<br>ABI StepOne Real-Time PCR System for use in library QC<br>PerkinElmer Sciclone NGSx Automated Library Prep Workstation<br>llumina HiSeq 2500
Data analysis	All downstream bioinformatic analysis was completed in R. Libary-size normalization, correction, t-SNE analyses, and clustering of cells was performed using the Seurat R package. Differential gene expression analysis and gene set enrichment analysis was performed using the MAST R package.<br>For integration site analysis PAIR (no version information available) (Paired End Read Merger): https://sco.h-its.org/exelixis/web/software/pear/<br>Needleman-Wunsch Alignment Tool (no version information available): https://www.ebi.ac.uk/Tools/psa/emboss_needle/nucleotide.html<br>GraphPad Prism version 7.03 for Windows (GraphPad Software, USA)<br>Custom Python scripts and publicly available R packages for analysis and display previously published in PMID 29093179 with all code sources cited and deposited in GitHub. Custom code links will be provided at the time of publication.

For manuscripts utilizing custom algorithms or software that are central to the research but not yet described in published literature, software must be made available to editors/reviewers. We strongly encourage code deposition in a community repository (e.g. GitHub). See the Nature Research guidelines for submitting code & software for further information.

Data

Policy information about availability of data

All manuscripts must include a data availability statement. This statement should provide the following information, where applicable:

- Accession codes, unique identifiers, or web links for publicly available datasets
- A list of figures that have associated raw data
- A description of any restrictions on data availability

TCR-seq data is provided as a Source Data file. scRNA-seq data are available at National Center for Biotechnology Information Gene Expression Omnibus (NCBI GEO) under accession number GSE125881. Integration site data are available at National Center for Biotechnology Information Sequence Read Archive (NCBI SRA) under accession number PRJNA589633. There are no restrictions on data availability. All data are available upon request.

Field-specific reporting

Please select the one below that is the best fit for your research. If you are not sure, read the appropriate sections before making your selection.

☒ Life sciences ☐ Behavioural & social sciences ☐ Ecological, evolutionary & environmental sciences

For a reference copy of the document with all sections, see nature.com/documents/nr-reporting-summary-flat.pdf

Life sciences study design

All studies must disclose on these points even when the disclosure is negative.

Sample size	No sample size calculations were performed for TCR-seq, integration site analysis, or single-cell RNA-seq experiments.
Data exclusions	In the single-cell RNA-seq experiments, cells with unique gene counts less than 200, a percentage of mitochondrial genes greater than 20% or greater than 40,000 UMIs were removed as low quality cells. In total, 2,340 cells were excluded. No data was excluded from integration site analysis.
Replication	In the single-cell RNA-seq experiments, differential gene expression analyses and gene set enrichment analyses between time points were performed within each patient. Only genes and gene sets that were statistically significantly in all patients were reported. A total of 7 biological replicates and 2 technical replicates within each sample were performed for integration site analysis.
Randomization	Randomization was not relevant to this study, though all indexed samples were pooled and sequenced in the same flow cell to reduce batch effects.
Blinding	Investigators were not blinded to the analysis.

Reporting for specific materials, systems and methods

We require information from authors about some types of materials, experimental systems and methods used in many studies. Here, indicate whether each material, system or method listed is relevant to your study. If you are not sure if a list item applies to your research, read the appropriate section before selecting a response.

Materials & experimental systems

n/a	Involved in the study
☐	☒ Antibodies
☒	☐ Eukaryotic cell lines
☒	☐ Palaeontology
☒	☐ Animals and other organisms
☐	☒ Human research participants
☒	☐ Clinical data

Methods

n/a	Involved in the study
☒	☐ ChIP-seq
☐	☒ Flow cytometry
☒	☐ MRI-based neuroimaging

Antibodies

Antibodies used

The following flow cytometry antibodies used in this study are commercially available.

AF488 anti-human CD160 antibody (Clone BY55, BD, Catalog # 562351)
PerCpe710 anti-human LAG3 antibody (Clone 3DS223H, eBioscience, Catalog # 46-2239-42)
A700 anti-human CD3 antibody (Clone UCHT1, BD, Catalog # 557943)
BV421 anti-human PD-1 antibody (Clone EH12.1, BD, Catalog # 562516)
Super bright 600 anti-human KLRG1 antibody (Clone 13F12F2, eBioscience, Catalog # 63-9488-42)
BV711 anti-human TIM3 antibody (Clone 7D3, BD, Catalog # 565567)

FITC anti-human CD4 antibody (Clone RPA-T4, BD, Catalog # 555346)
BV750 anti-human CD4 antibody (Clone SK3, BD, Catalog # 566355)
APC anti-human CD8 antibody (Clone RPA-T8, BD, Catalog # 555369)
BUV496 anti-human CD8 antibody (Clone RPA-T8, BD, Catalog # 564804)
BUV805 anti-human CD45 antibody (Clone HI30, BD, Catalog # 564914)
PE anti-human TIGIT antibody (Clone MBSA43, eBioscience, Catalog # 12-9500-42)
PE-Cy7 anti-human 2B4 antibody (Clone C1.7, Biolegend, Catalog # 329520)
PE Streptavidin (BD, Catalog # 554061)

Validation	Commercially available flow cytometry antibodies have been validated by the vendor.

Human research participants

Policy information about studies involving human research participants

Population characteristics	Patient characteristics are listed in Supplementary Data 1.
Recruitment	We studied a subset of adult patients with relapsed and refractory B cell ALL, NHL, or CLL who received CD19-specific CAR-T cells in a phase 1 clinical trial (NCT01865617) and had sufficient numbers of CD8+ CAR-T cells in the blood after infusion at the early (day 7-14) and late (day 26-30) time points for TCRB repertoire analysis as well as at the very late (day 83-112) time point for single-cell transcriptome analysis.
Ethics oversight	Fred Hutchinson Cancer Research Center Institutional Review Board

Note that full information on the approval of the study protocol must also be provided in the manuscript.

Flow Cytometry

Plots

Confirm that:

☒ The axis labels state the marker and fluorochrome used (e.g. CD4-FITC).

☒ The axis scales are clearly visible. Include numbers along axes only for bottom left plot of group (a 'group' is an analysis of identical markers).

☒ All plots are contour plots with outliers or pseudocolor plots.

☒ A numerical value for number of cells or percentage (with statistics) is provided.

Methodology

Sample preparation	Cryopreserved aliquots of the infusion product or peripheral blood mononuclear cells isolated from the blood were thawed in RPMI containing 50 U/ml of benzonase.
Instrument	BD Aria 2 flow sorter; BD FACSymphony flow cytometer
Software	FlowJo Software
Cell population abundance	The purity of sorted cells was > 90% as tested by running post-sort samples on the flow cytometer. Single-cell RNA-seq data further confirmed the purity of sorted cells based on mRNA expression of CD3 subunits, CD8A, and scFv.
Gating strategy	For all flow cytometry experiments, the lymphocyte population was pre-gated based on forward and side scatter measurements. Singlets were gated using side scatter height and width measurements. CD8+ CAR-T cells were gated as CD8+ CD4-/EGFRt+ events.

☒ Tick this box to confirm that a figure exemplifying the gating strategy is provided in the Supplementary Information.
